# Cocoa Polyphenols and Inflammatory Markers of Cardiovascular Disease

**DOI:** 10.3390/nu6020844

**Published:** 2014-02-21

**Authors:** Nasiruddin Khan, Olha Khymenets, Mireia Urpí-Sardà, Sara Tulipani, Mar Garcia-Aloy, María Monagas, Ximena Mora-Cubillos, Rafael Llorach, Cristina Andres-Lacueva

**Affiliations:** 1 Biomarkers Research Program, Biochemistry Department, College of Science, King Saud University, P.O. Box 2455, King Abdullah road, Riyadh 11451, Saudi Arabia; E-Mail: nasiruddin2006@gmail.com; 2 Biomarkers and Nutritional & Food Metabolomics Research Group, Department of Nutrition and Food Science, XaRTA, INSA, Campus Torribera; INGENIO-CONSOLIDER Program, Fun-C-Food CSD2007-063, Ministry of Science and Innovation, Faculty of Pharmacy, University of Barcelona, Av Joan XXIII, s/n, Barcelona 08028, Spain; E-Mails: murpi@ub.edu (M.U.-S.); sara.tulipani@gmail.com (S.T.); margarcia@ub.edu (M.G.-A.); ximenamora@ub.edu (X.M.-C.); rafallorach@ub.edu (R.L.); candres@ub.edu (C.A.-L.); 3 Biomedical Research Institute (IBIMA), Service of Endocrinology and Nutrition, Hospital Complex (Virgen de la Victoria), Campus de Teatinos s/n, University of Málaga, Malaga 29010, Spain; 4 Institute of Food Science Research (CIAL), CSIC-UAM. C/Nicolás Cabrera 9, Campus de Cantoblanco, Madrid 28049, Spain; E-Mail: m.monagas@csic.es

**Keywords:** cocoa polyphenols, bioavailability, inflammation, CVD

## Abstract

Epidemiological studies have demonstrated the beneficial effect of plant-derived food intake in reducing the risk of cardiovascular disease (CVD). The potential bioactivity of cocoa and its polyphenolic components in modulating cardiovascular health is now being studied worldwide and continues to grow at a rapid pace. In fact, the high polyphenol content of cocoa is of particular interest from the nutritional and pharmacological viewpoints. Cocoa polyphenols are shown to possess a range of cardiovascular-protective properties, and can play a meaningful role through modulating different inflammatory markers involved in atherosclerosis. Accumulated evidence on related anti-inflammatory effects of cocoa polyphenols is summarized in the present review.

## 1. Introduction

Of an estimated 17.3 million deaths globally from all causes in 2008, cardiovascular disease (CVD) accounted for 30% [1]. The cardiovascular epidemic is rapidly advancing in the world. It has been projected that by 2030 nearly 23.6 million people will die from cardiovascular disorders [2,3]. However, the majority of these deaths caused by CVD are preventable [4,5]. Epidemiological and clinical studies have shown that lifestyle modifications such as nutrition and exercise are initial protective measures to reduce the risk of CVD [6,7,8,9]. The consumption of plant-derived food, such as whole grains, fruits, and vegetables, has been recognized as one of the principal preventive factors in the risk of all-cause and CVD mortality [10,11,12]. Moreover, the absolute quantity and frequency of fruit and vegetable intake have been associated with lower CVD risk and, therefore, with lower CVD mortality [13,14]. The mechanisms by which fruit and vegetables exert their protective effects are not entirely clear, but experimental and population studies [15,16] have indicated that plant polyphenols are principal mediators. Numerous scientific reports accumulated over recent decades suggest that plant polyphenols may exert their activities on the antioxidant system, signaling and transcription pathways, thus affecting principal mechanisms involved in cardiovascular events, such as systemic inflammation, lipid metabolism, hemostatic and vascular events, and immune response [17,18,19,20,21,22,23].

Cocoa products are among the richest sources of polyphenols in our diets. Nowadays cocoa has become a widely consumed food component, with growing demand across the world [24]. Interest in the biological activities of cocoa (*Theobroma cacao*) polyphenols has increased steadily, since the first studies on the positive link between plant food polyphenol consumption and health outcomes were reported. Cocoa has the highest flavanol content of all foods on a per-weight basis and is a significant contributor to the total dietary intake of flavonoids [25,26]. Depending upon geographical origins and plant varieties, the total polyphenol content of cocoa ranges from 40.0 mg GAE/g (GAE; gallic acid equivalent) to 84.2 mg GAE/g [27,28,29,30]. However, portions in chocolate, the most commonly consumed processed cocoa product, are significantly lower, with levels of 1.7–8.4 mg/g reported in dark chocolate and even lower levels of 0.7–5 mg/g in milk chocolate [31,32].

Several population studies have reported on the inverse association between cocoa intake (e.g., chocolate) and CVD mortality [33,34]. A number of recent meta-analyses of intervention studies have demonstrated that there is substantial evidence that cocoa consumption affects multiple cardiovascular risk factors such as blood pressure [35], insulin resistance [36], lipid profiles [37], and flow-mediated vascular dilatation (FMD) [38]. On the other hand, there are numerous *in vitro* and *in vivo* experimental data supporting the notion that polyphenols may mediate these beneficial effects of cocoa [39,40,41].

Nowadays, it has become more evident that inflammatory mediators play a key role in the pathology of atherosclerosis, starting from the initial phases of leukocyte recruitment, and finishing with the eventual rupture of the vulnerable atherosclerotic plaque [42,43,44]. Therefore, atherosclerosis and cardiovascular pathologies caused by it are readily recognized and treated as inflammatory diseases [45]. Practically all cardiovascular risk factors are to a different extent linked to inflammation, while inflammation itself is recognized as a cardiovascular risk factor [46,47].

The participation of dietary polyphenols in the modulation of inflammation and how this could contribute to the reduction of cardiovascular risk was recently discussed [48]. There is a growing body of evidence on the anti-inflammatory activities of cocoa polyphenols. The protective role of cocoa in CVD inflammation has been considered in a number of human intervention studies, and numerous *in vitro* studies have also been carried out, led by an interest in disclosing the mechanisms and identifying the agents responsible for the anti-inflammatory cardio-protective activities of cocoa. Accordingly, polyphenols have been proposed as principal anti-inflammatory mediators. In this review, we aim to summarize these findings in order to assess the effects of cocoa polyphenols on cardiovascular-related inflammation.

## 2. Cocoa Polyphenols

**Figure 1:**
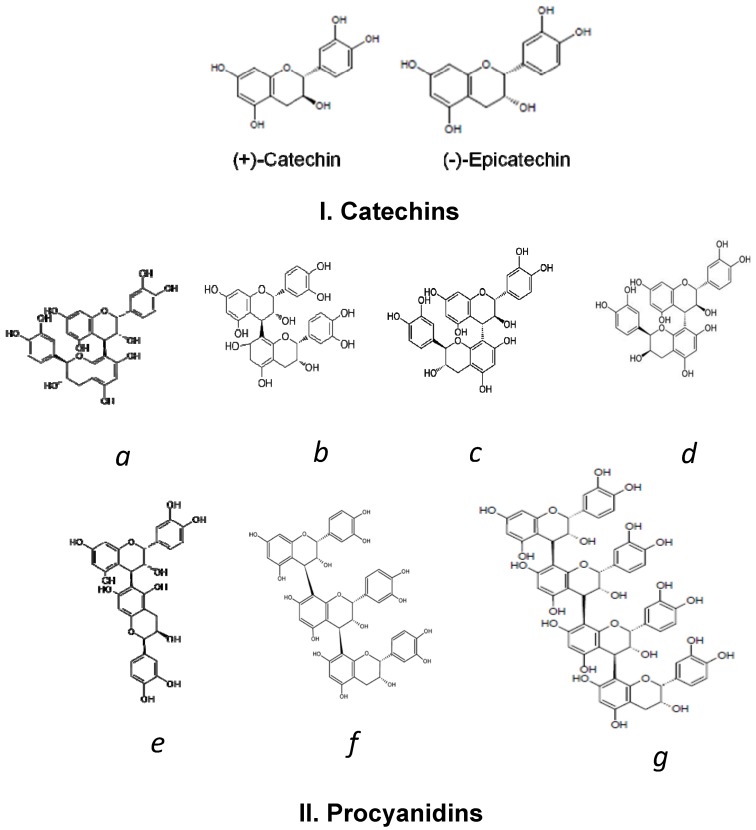
(**I**) Main cocoa flavanol monomers: (+)-catechin; (−)-epicatechin; (**II**) Main cocoa flavanol polymers: (**a**–**e**)—dimers: procyanidin B1, B2, B3, B4, B5, respectively; (**f**)—trimer: procyanidin C1; (**g**)—tetramer: procyanidin D.

Polyphenols in cocoa beans could contribute to about 12%–18% of the dry weight [49], making them practically inedible due to the bitterness and astringency [50]. Wollgast and Anklam [51] reported that catechins, anthocyanins, and proanthocyanidins constitute about 37%, 4% and 58% of cocoa bean polyphenols, respectively. Flavanols are the most important class of cocoa polyphenols [51,52]. They are presented by monomers ((+)- and (−)-isomers of catechin and epicatechin and their derivatives) and build-up of (epi)catechin subunit polymers (proanthocyanidins) [53]. Phenolic acids, flavonols and their glycosides, some stilbenes, simple phenols, and isocoumarins are also present in minor amounts [54,55,56]. Cyanidin-3-α-l-arabinoside and cyanidin-3-β-d-galactoside have been reported to be principal anthocyanins of cocoa, however, mainly in unprocessed fresh beans [51]. (−)-Epicatechin constitutes about 35% of the total phenolic content of cocoa beans, while other catechins, such as (+)-catechin, (+)-gallocatechin, and (−)-epigallocatechin, are present in smaller quantities. Dimers (B1, B2, B3, B4 and B5), trimers (C1), and oligomers (tetramer D) of flavan-3,4-diols, linked by 4→8 or 4→6 bounds (B-type linkage), represent the main cocoa polymers, procyanidins [53,57,58]. The structures of the main cocoa polyphenols, monomeric and polymeric flavanols, are shown in Figure 1. Some other procyanidins have been identified in smaller amounts in fresh cocoa beans: dodecamer and three A-type (2→7 or 2→5 along with 4β→8 linkage) procyanidin dimmers [53,59] probably derive from oxidative conversion of B-type procyanidins [60,61]. In general, unfermented (fresh) cocoa beans contain flavanols with a varied range of polymerization, from monomers up to decamers [62].

The polyphenol content of cocoa could vary up to 4-fold depending on different varieties [63] and origins [64]. In addition to the above factors, cocoa beans undergo several steps of primary and secondary processing due to which the ratio and types of polyphenols found in cocoa beans are different from those found in the finished products [65]. For instance, the processes of fermentation and alkalization reduce the polyphenol content [63,66,67,68] and, consequently, the antioxidant activity of cocoa beans [32,67]. Thus, (−)-epicatechin and (+)-catechin decrease depending on the extent of cocoa bean fermentation: unfermented, partly fermented or fully fermented. In addition, high temperatures and long duration of processing also decrease the polyphenol content [51,68,69]. Some special methodological adjustments could be applied in order to prevent cocoa polyphenols from oxidation during different stages of cocoa processing, increasing their content in a final product [70,71].

Semi-finished products such as cocoa liquor, and cocoa butter and cocoa powder derived from it, which are manufactured during secondary processing, are usually used in manufacturing final cocoa products (e.g., cocoa powder and chocolate) [72]. Sometimes unprocessed cocoa bean powder is introduced, mainly in order to enrich the polyphenol content of the final product [50,73]. The content of polyphenols in the final product is defined by the amount and type of cocoa components. Thus, cocoa powder is shown to have higher polyphenol content than cocoa butter [39,74], since it is mainly the low-fat solid component of cocoa nibs. Non-fat cocoa solids (NFCS) are considered to represent the total phenolic content in cocoa products [75]. Based on NFCS, cocoa powder represents the highest phenolic content (72%–87%), followed by baking chocolate (45%–49%), dark chocolate (20%–30%), semi-sweet chocolate (15%–19%), and milk chocolate (5%–7%) [76]. The quantitative determination of polyphenol content in a cocoa product can be assessed by using either total polyphenols [31] or the measurements of (−)-epicatechin and (+)-catechin [26], and also B-type procyanidin contents [73]. Based on weight in grams, (−)-epicatechin and (+)-catechin content follows a decreasing order, cocoa powder > dark chocolate > milk chocolate [77], while each content could be widely variable. For example, for cocoa powder it can range from 116.02 to 730.26 μg/g for (−)-epicatechin, and from 81.40 to 447.62 μg/g for (+)-catechin, the total monomeric content ranging from 182.84 to 1066.13 μg/g [68]. In general, according to the polyphenol database Phenol-Explorer [78], the mean values of (−)-epicatechin and (+)-catechin are about 158 and 107, 70 and 20, and 15 and 5 mg/100 g FW in cocoa powder, dark chocolate, and milk chocolate, respectively. However, the amount of consumed cocoa polyphenols would also depend on serving size, thus, in a normal diet, dark chocolate would provide more polyphenols than other cocoa products [39].

As a consequence of whole processing, cocoa-derived products mainly contain less bioavailable (−)-catechin enantiomer, in contrast to cocoa beans and most foods that contain (+)-catechin [79,80]. Analyses of polyphenols have shown a relative abundance of oligomers and individual components in cocoa liquor, which follows the order: monomers > trimers > dimers > tetramers and epicatechin > procyanidin B2 > procyanidin C1 > catechin, respectively [81]. The trend in cocoa powder was different and followed the order: monomers > dimers > trimers > tetramers and epicatechin > catechin > procyanidin B2 > procyanidin C1, respectively [81]. Trace amounts of gallocatechin and epigallocatechin [55] and quercetin aglycone as well as some quercetin glycosides have been identified in cocoa liquor and cocoa powder [56,82]. Some additional flavonoids, such as naringenin, luteolin, apigenin and some glycosides of these compounds, and quercetin-glucuronide were also reported [68,77].

On the whole, during cocoa processing the polyphenol content is reduced more than ten times [74,83], with the undesirable bitter and astringent taste diminishing sometimes in final cocoa products. Cocoa food products existing on the market are characterized by highly diverse polyphenol content, mainly due to the differences in cocoa processing and product manufacturing, in addition to intrinsic geographical and genetic plant divergence [61,73,77,84]. Epidemiological and clinical studies on the health benefits of cocoa polyphenols should account for this variability in addition to other aspects, such as the bioavailability of putatively bioactive cocoa polyphenols, which we will discuss in the following section.

## 3. Bioavailability of Cocoa Polyphenols

In order to provide conclusive evidence for the effectiveness of cocoa polyphenols in disease prevention and human health improvement, it is essential to determine the nature and distribution of these compounds in our diet. In addition, the bioavailability of the ingested cocoa polyphenols will circumscribe organism exposure to these putatively bioactive compounds, affecting a magnitude of related health outcomes. There are a number of factors influencing the bioavailability of cocoa polyphenols, starting from their dietary consumption and finishing with their fate in the human organism.

The daily consumption of cocoa catechins and procyanidins depends on cultural and regional dietary habits [75]. It has been estimated that in some regions (e.g., Belgium/Luxemburg) cocoa product consumption could reach up to 6 kg in cocoa beans/person per annum, the world average being 0.55 kg/person per annum [85]. The majority of intervention and cross-sectional studies regarding the health effects of cocoa have been conducted using cocoa beverages or chocolate as the most habitually used cocoa products [86], therefore bioavailability and intervention studies have also been focused mainly on these cocoa products. The protagonists of these studies are the major cocoa flavanols, epicatechin and procyanidins, since they have so far been foreseen as the principal bioactive cocoa polyphenols due to both their abundance and their relevance to biological activities and physicochemical structures [49,87,88].

Upon ingestion of cocoa products in doses close to habitual diet monomeric and polymeric cocoa flavanols are rapidly absorbed. Thus, it was shown that procyanidins and monomers could be detected in plasma as early as 0.5 h and maximal plasma concentration of these compounds was reached at about 2 h after ingestion [89,90,91,92,93,94,95,96,97,98,99,100]. The plasmatic T_max_ of detected cocoa flavanols did not normally exceed 3 h, and their elimination from plasma in most cases was already achieved 6 h after cocoa consumption [94,95,96]. However, some epicatechin metabolites could have a delayed appearance and could remain in systemic circulation for up to 24 h, suggesting their intestinal microbiota catabolic origin [97,98]. Rapid plasmatic appearance suggests that the detected polyphenols are mainly absorbed in the small intestine. This was ratified by the data coming from *in vitro* and *in vivo* bioavailability studies on catechins and procyanidins [99,101,102]. However, absorption of flavan-3-ols in the small intestine is not complete, and depends not only on flavanol chemistry, but also on their structural isomerism and stereoisomerism [103]. Therefore, epicatechins and catechins were shown to have different levels of absorption [93,94], and plasma concentration of (+) and (−) forms of catechin were found to be different after cocoa product consumption [79,104]. The level of absorption, furthermore, depends on the range of polymerization, which will also limit their bioavailability [49]. *In vitro* studies suggested that flavanols only up to trimers were able to pass through the small intestine [101,102]. In humans, only traces of procyanidin B2 were detected in human plasma after ingestion of cocoa products [93,94]. More complex flavanols appeared to be stable under acidic conditions mimicking stomach digestion [105], so that, being unabsorbed in the small intestine, they are transited to the large intestine, where they are subjected to catabolic activities of intestinal microbiota [106].

Under absorption in the gastrointestinal tract, cocoa flavanols (monomers) are recognized by the organism as xenobiotics and are extensively transformed into various metabolites [107]. (−)-Epicatechin in its sulfate, glucuronides or methyl conjugated forms were the main forms representing about 33%, 28%, and 33% of total epicatechin metabolites in human plasma and urine [97], the most relevant being (−)-epicatechin-3′-β-d-glucuronide, (−)-epicatechin 3′-sulfate, 3′-*O*-methyl-(−)-epicatechin sulfates [97,108]. In fact, epicatechin aglycone was undetectable both in plasma and urine according to recent findings, where advanced methods were applied [97,108].

Due to the gastrointestinal and hepatic metabolism, the conjugated metabolites are destined to be rapidly eliminated from the human body. Cocoa flavanols, absorbed in the small intestine, are normally cleared form the body over 24 h, like the majority of dietary polyphenols. On the other hand, a large proportion of unabsorbed flavanols, e.g., polymers and monomers, are subjected to colonic microflora catabolic activities [106,109,110]. Consequently, low-molecular-weight microbiota catabolites of these flavanols are absorbed from the colon, belatedly emerging in systemic circulation as phase II conjugates [106,107,111]. Valerolactones and valeric acids were reported to be first-step microbiota-derived catabolites [106,112] of unabsorbed small intestine epicatechin and procyanidins [112,113,114,115], whereas various phenolic acids were identified as intermediate and last-step products of microbial flavanol catabolism [106,111,113,116]. On the other hand, a part of unabsorbed flavanols can accumulate in the low digestive tract and are finally excreted from the human body with faeces [106,117,118]. Plasmatic concentrations vary widely among reported flavanol microbiota metabolites, some of them reaching micromolar levels in response to cocoa polyphenol consumption [114]. The studies in human urine have not only indicated the bioavailability but also demonstrated the importance of the phase II conjugated metabolites and some colonic microbiota metabolites as promising biomarkers of cocoa consumption [119,120,121], justifying their application for dietary compliance control in cocoa intervention studies along with hydrolyzed epicatechin [114,122,123,124].

Recently, the scientific community has become aware that the microbe-derived metabolites of polyphenols represent a large proportion of dietary polyphenol intake [106,125,126,127,128], impacting on their bioavailability and potentially exhibiting some bioactive effects [129,130,131,132]. Moreover, regular consumption of polyphenol-rich cocoa could in turn influence the colonic bacterial population and metabolic activities [133], enlarging inter-individual flavanol bioavailability variation [134]. For example, a significant difference in bacterial metabolite profiles between regular cocoa product consumers and non-consumers was reported in response to dark chocolate intervention [135]. Nowadays, microbial cocoa metabolites are being reconsidered with regard to their health-related bioactivities, including those related to CVD inflammation, which we will address later in the corresponding section.

The interaction between different nutrients, the food matrix and texture are one of several important factors that affect the bioavailability of cocoa polyphenols. The effect of milk, the most commonly used food element in cocoa-formulated products, on the bioavailability of both primary cocoa polyphenols and their host and microbiota metabolites has been studied with different cocoa products [96,113,136,137,138,139,140]. However, conclusions on this issue have remained controversial. In addition, carbohydrates and proteins [141,142,143], along with the complex food matrix and cocoa product physical stage (liquid *vs*. solid) [144,145], were also considered to interfere in the mechanism of flavanol uptake, affecting the bioaccessibility and bioavailability of cocoa polyphenols in human subjects.

Although in the majority of the intervention studies the doses of cocoa products were close to high rather than average in habitual consumption, systemically circulating (plasmatic) concentrations of cocoa flavanols were reported to be relatively low—from nano- to micro-molar range for epicatechin and not always detected nano-molar concentrations for procyanidins [95,98,100,146]. Low maximum concentration (C_max_), a short half-life, extensive metabolic conjugation and rapid excretion, all add up to relatively low cocoa polyphenol bioavailability, compromising their relevance for health effects and bioactivities reported in *in vitro* and *in vivo* mechanistic studies [147]. On the other hand, there are data showing that sustained chronic consumption of cocoa products can lead to a relatively low but significant accumulation of cocoa polyphenols in human plasma [148], sufficient to exert some health-relevant bioactivity. These data support the idea that high doses of cocoa polyphenols might not always be needed to manifest biological activities, and the time of interaction could also be important.

The absorption of cocoa polyphenols, characterized as epicatechin 24 h urinary recovery data, was considered to be poor, reaching not more than 30% of the total ingested (−)-epicatechin both with chocolate and with cocoa powder [97,98]. Some studies reported much lower recoveries [96,103], especially when the cocoa polyphenol doses were closer to habitual dietary consumption and cocoa products were co-administrated with milk [96]. The participation of procyanidins in epicatechin bioavailability due to catabolic activities of colonic microflora is still being questioned due to the controversy in reported data [108,115]. Data on cocoa polyphenol absorption did not account for microbial-derived metabolites. So far, addressing this issue has proved challenging, since the majority of identified microbiota flavanol catabolites are not epicatechin specific and could also be related to other dietary polyphenols and/or unrelated microbial activities [106].

Although levels of circulating cocoa polyphenol metabolites appeared to be dose dependent [95,100], high inter-individual variability, mainly defined by individual metabolic phenotypes [149,150], was reported in practically all bioavailability studies. On the other hand, large inter-individual differences in colonic flavanol bioconversion, attributed to the individual colonic microbiota composition, are also expected to have an impact on variation in the bioavailability of cocoa polyphenols [127,134]. Such variability is difficult to control on the population level due to the nature of its matter; therefore, it still remains one of the main concerns in interventional studies regarding polyphenols’ health-benefiting activities, as it compromises the accurate estimation and correct interpretation of studied outcomes.

## 4. Cocoa Polyphenols and CVD Inflammatory Markers

Evidence from several epidemiological studies has demonstrated the favorable association of cocoa and its derived products with a lower risk of CVD mortality [33,40,151]. Thus, the effect of high cocoa consumption in Kuna Indians who lived in their indigenous islands in Panama has been related to lower blood pressure and low mortality rates as compared to Kunas emigrated to the urban areas of Panama City, revealing its favorable effect [152]. In the Dutch Zutphen Study, in a cohort of elderly men, high cocoa intake was related to lower blood pressure and was inversely associated with cardiovascular and all-cause mortality [33]. In Women’s Health Study, chocolate intake was also found to be inversely associated with cardiovascular mortality, along with other foods rich in flavonoids, such as some fruits and red wine [153].

Although the mechanism for the beneficial effects of cocoa and its derived products still remains to be fully elucidated, the potential biological role of flavanols has been suggested by various human interventions [36,38,39,40] and *in vitro*/*in viv*o mechanistic studies [39,40,41]. So far, among the most relevant impacts of cocoa polyphenols on cardiovascular health should be highlighted their effect on the most significant markers of CVD: oxLDL [124,154,155], lipid profile [124,154,156], blood pressure [35,157,158], nitric oxide [52,159], hemostasis [91,148,160] and endothelial dysfunction [159,161,162].

Thus, it is well known that alteration in plasma cholesterol levels (LDL-c and HDL-c) is related to the progression of atherosclerosis and CVD [163]. Dietary medium-term intervention with cocoa powder in mild hypercholesterolemic subjects showed significantly lower levels of LDL-c [164], while the level of HDL-c was found to be increased in normo- and mild hyper-cholesterolemic subjects after dark chocolate or cocoa powder consumption [154,164,165]. A recent study in women affected by normal weight obese syndrome, which is characterized by a higher risk of cardiovascular morbidity and mortality, suggested that regular consumption of dark chocolate had favorable effects on HDL-c, lipoprotein ratios and inflammation markers [166]. Oxidized LDLs play a crucial role in the progression of atherosclerosis [167]. There are several studies that have established oxidized low-density lipoprotein (oxLDL) as a useful marker for cardiovascular diseases [168,169,170,171]. Cocoa polyphenols have been shown to decrease the oxidation of LDL in *in vitro* studies [172,173]*.* In addition, intervention studies have demonstrated that isolated LDLs are less prone to *in vitro* oxidation after the consumption of various cocoa products [154,155,174,175,176]. Moreover, a recent study including 42 high-risk human subjects reported a significant decrease in plasma oxLDL levels in addition to a significant increase in plasma HDL-c concentration after chronic cocoa consumption [124]. The role of cocoa polyphenols has also been investigated for their effect on vascular systems, which could lead to reduced risk of CVD. The main targets include nitric oxide (NO) concentration and endothelial function, along with decreased susceptibility of LDL to oxidation and inhibition of platelet activation and aggregation [155,177,178]. In numerous studies, which were recently reviewed [179], both acute and sustained consumption of flavanol-containing cocoa products were reported to have a dose-dependent beneficial effect on endothelial function via an improvement in FMD. An increase in the plasma nitric oxide (NO) concentration has been shown in healthy subjects after the consumption of cocoa beverages containing different contents of flavanols. This change in plasma NO-modulated FMD indicates an association between increased bioavailability of NO and improved endothelial function [180]. Cocoa polyphenols from dietary sources may also improve endothelial functions by augmenting NO-synthase activity [52,159,181,182], leading to a decrease in systolic and diastolic blood pressure [162,183,184]. Platelet aggregation occurs during the initiation of coronary thrombosis and several studies support evidence about the potent activity of cocoa polyphenols as inhibitors of platelet aggregation and adhesion, thus reducing clot formation [185,186].

Cardiovascular pathology is accompanied by chronic low-intensity inflammation, involving the participation of a variety of cells (endothelial, smooth muscular, monocytes, lymphocytes and platelets), adhesion molecules (selectins, integrins and immunoglobulin superfamily molecules), cytokines (pro-inflammatory and anti-inflammatory), chemokines, growth factors and enzymes (metalloproteases, cyclooxygenases and lipoxygenases) [187]. These inflammatory mediators appear to play a key role in each step of atherogenesis, starting from the initial phases of leukocyte recruitment, to the eventual rupture of the vulnerable atherosclerotic plaque [44]. Some of these key players are often used in the evaluation of a grade of pathological changes or response to treatment, usually referring to them as CVD-related inflammatory markers [188]. Apart from this, inflammation itself is considered a risk factor for CVD [187,189,190]. Therefore, circulating levels of the inflammatory markers could also reflect the current inflammatory state of the individuals, exhibiting their risk status in respect of CVD. The accumulated evidence on the health benefits of cocoa for disorders of a chronic inflammatory nature, such as CVD, prompted the need to focus on the association between bioactive cocoa polyphenols and inflammatory mediators of CVD, with the purpose of identifying and recognizing factors involved and mechanisms underlying this interaction.

### 4.1. Human Studies

Up until now, only one epidemiologic study has reported on the relationship between cocoa consumption and inflammation. Thus, a large cohort study involving men and women randomly recruited from the general population demonstrated a J-shaped relationship between dark chocolate consumption and serum hs-CRP, a CVD inflammatory marker. Those subjects who usually consumed up to 1 serving (20 g) of dark chocolate every 3 days had significantly lower serum hs-CRP concentrations than non-consumers [191]. Most of the evidence on the possible interaction between cocoa polyphenols and CVD-related inflammation was obtained in clinical intervention studies involving human subjects. The human intervention studies evaluating the effect of cocoa polyphenols on inflammatory mediators of CVD are presented in Table 1.

As well as the above-mentioned epidemiological study, CRP (C-reactive protein), a biomarker strongly associated with coronary heart disease and inflammation [192], was evaluated in several clinical trials with cocoa (Table 1). However, only a few of these studies reported on changes in CRP levels due to cocoa consumption. Thus, one of the first intervention trials performed by Mathur [176] and Kurlandsky [193] reported no changes in hs-CRP circulation in healthy subjects due to medium-term intervention with dark chocolate. However, a decrease in the CRP systemic levels of healthy subjects was observed after short- and medium-term daily consumption of cocoa beverages in two recent studies [194,195]. The decrease in circulating CRP was shown to be linear in response to the polyphenol content of the consumed cocoa beverages, according to the data from the latest study [195]. It is worth noting that both studies had crossover designs with well-matched treatment controls (adjusted for other important cocoa bioactive compounds, such as theobromine and caffeine), which reinforce the evidence on the role of cocoa polyphenols in this interaction. A short-term intervention study reported on specific gender changes in hs-CRP upon dark chocolate consumption observed in healthy female but not male volunteers [196]. However, these findings should be confirmed in a bigger, controlled matched intervention on a well-recruited female population to avoid any impact of hormonal status during the menstrual cycle on systemic inflammation [197]. In contrast to crossover studies on healthy subjects, no impact of cocoa consumption was observed on the hs-CRP level in subjects with compromised cardiovascular health in the few available studies [123,198,199]. Thus, in a medium-term crossover study with hypertensive pre-diabetic subjects, dark chocolate consumption was compared to white chocolate with an impact on certain CVD markers, including circulating levels of CRP. Although authors reported a beneficial effect of medium-term consumption of dark chocolate on vascular function, insulin sensitivity, and BP in hypertensive patients, no effects were observed on the hs-CRP level [199]. No change in hs-CRP levels was detected either in subjects at high cardiovascular risk in another crossover study after chronic cocoa powder in milk intake [123]. A parallel arm intervention study [198], involving patients with coronary artery disease, reports that over a 6-week period, flavanol-rich cocoa does not modify vascular function in patients with established CAD. Along with vascular functions evaluated postprandially (90 min) and after chronic consumption (3 and 6 weeks), circulating hs-CRP was evaluated. However, it was not affected either by acute or chronic consumption of cocoa-derived products.

**Table 1:** Human intervention studies considering the relationship between cocoa polyphenols and cardiovascular disease (CVD)-related inflammatory markers (studies are presented in chronological order). Ref. = Reference.

Intervention				Comparison	Population		Markers of Dietary Compliance	Cocoa Consumption Impact	Ref.
Cocoa Source (dose)	Type (time)	Study Design	Polyphenol Content		*N*	Subjects Status			
High- *vs*. Low-procyanidin chocolate (37 g/dose)	AI	CO	High-procyanidin chocolate: 148 mg tot. Pr	H *vs*. L	10	Healthy	2 h plasma (BM) 6 h plasma (BM)	2 h postprandial:	[89]
								↓leukotriene/prostacyclin ratio	
			Low-procyanidin chocolate: 3.3 mg tot. Pr					↓leukotrienes (C_4_ + D_4_ + E_4_)	
								↑prostacyclins	
Dark chocolate + cocoa beverage (36.9 g + 30.95 g/day)	MTI (6 weeks)	NC	Chocolate (daily):	Before/After	25	Healthy	Plasma (tot. PPh)	No effect: IL-1beta, IL-6, TNF-α, hs-CRP, P-selectin	[176]
			168.3 mg Pr						
			Cocoa beverage (daily):						
			483.1 mg Pr						
Dark chocolate (41 g/day)	MTI (6 weeks)	PA	NA	Before/After DC *vs*. Ctrl group	10	Healthy	NA	↓ICAM-1	[193]
								No effect: VCAM-1 and hs-CRP	
Chocolate and cocoa beverage (48 g chocolate + 18 g cocoa beverage/day)	AI (90 min)	PA PA	Flavanol group (daily):	Before/After Flavanol *vs*. Placebo (Ctrl)	40	Coronary artery disease (CAD)	NA	No effect (acute or chronic): ICAM-1, VCAM-1, E- and P-selectins and hs-CRP	[198]
			444 mg flavanols						
			107 mg epicatechin						
	MTI (6 weeks)		Control group (daily):						
			19.6 mg flavanols						
			4.7 mg epicatechin						
Low- and High-flavanol cocoa beverages (36 g powder (18.8 g cocoa) per 240 mL W)	MTI (6 weeks)	PA	High-flavanol group:	Before/After H *vs*. L group	32	Postmenopausal hypercholesterolemic women	Fasting plasma (BM)	↓sVCAM-1(High-flavanol group) No effect: ICAM-1; E- and P-selectins	[200]
			446 mg tot. flavanols						
			Low-flavanol group:						
			43 mg tot. flavanols						
Dark chocolate 70% (100 g/day)	STI (7 days)	NC	Daily: 700 mg flavonoids	Before/After	28	Healthy	NA	↓hs-CRP (females only, *n* = 19)	[196]
								No effect: hs-CRP (common group)	
Dark chocolate (50 g/day × 2 times/day)	MTI (15 days)	CO	Dark chocolate (daily):	DC *vs*. WC (Ctrl)	19	Hypertensive prediabetic	NA	No effects: hs-CRP	[199]
			1008 mg tot. PPh						
			110.9 mg epicatechin						
			36.12 mg catechin						
			White chocolate(daily):						
			0.13 g tot. PPh						
			0.04 mg catechin						
Cocoa beverage (31 g/150 mL W × 2 times/day	MTI (2 weeks)	CO	Flavanol beverage (daily):	Before/After Flavanol *vs*. Control beverage (Ctrl)	20	Hypertensive	Fasting plasma (BM)	No effect: TNF-α, IL-6, MCP-1, *E*-selectin, VCAM-1, and ICAM-1	[201]
			451 mg tot. PPh						
			57 mg epicatechin						
			31 mg catechin						
			338 mg Pr						
			Control beverage (daily):						
			14 mg tot. PPh						
			1 mg epicatechin						
			4 mg catechin						
			8 mg Pr						
Cocoa powder (20 g/250 mL M × 2 times/day)	MTI (4 weeks)	CO	Daily: 40.41 mg (+)-catechin	Cocoa (CM) *vs*. M (Ctrl)	42	CVD high risk	24 h urine (BM)	↓VLA-4, CD40, CD36 (monocytes) ↓P-selectin and ICAM-1 (serum) Non-significant changes: ↓VCAM-1 and MCP-1 No effect: hs-CRP, IL-6, E-selectin	[123]
			46.08 mg (−)-epicatechin						
			36.54 mg procyanidin B2						
			495.2 mg tot.PPh						
			425.7 mg tot.Pr						
High- and Low-cocoa flavanol beverage (NA)	MTI (4 weeks)	CO	High-cocoa flavanol (daily):	Before/After H *vs*. L	20	Healthy	Fasting plasma (BM) 24 h urine (BM)	↓CRP (High-cocoa flavanol group)	[194]
			494 mg tot. Flavanols						
			Low-cocoa flavanol (daily):						
			23 mg tot. flavanols						
Different flavanol content cocoa beverages *vs*. Control beverage (28 g cocoa powder in W × 2 times/day)	STI (5 days)	CO	Cocoa beverages (daily):	L, M, H *vs*. Control beverage (Ctrl)	20	Obese healthy	NA	↓CRP ↓IL-6 No effect: ICAM	[195]
			180 mg flavanols (Low)						
			400 mg flavanols (Medium)						
			900 mg flavanols (High)						
			Control beverage (daily):						
			30 mg flavanols						
Dark chocolate 70% (25 g ×2 times/day)	MTI (4 weeks)	NC	Daily: 2135 mg PPh	Before/After	20	Hypertensive (excess body weight)	NA	Non-significant changes:	[202]
								↓ICAM-1	
								↓VCAM-1,	
								↓E-selectin	
Dark chocolate 70% (100 g/day)	STI (7 days)	NC	444 mg/kg catechin	Before/After	15	Normal weight obese women	NA	↓IL-1Ra and No effect: IL-1α, IL-1β, IL-6, and TNF-α	[166]
			908 mg/kg epicatechin						
Cocoa product rich in fibre (15 g/200 mL M × 2 times/day)	MTI (4 weeks)	CO	13·9 mg/g soluble PPh	Cocoa *vs*. M (Ctrl)	24 20	Healthy and Hypercholesterolemic subjects	NA	↓IL-1β, IL-10	[156]
								No effect: IL-6, TNF-α, IL-8,	
								MCP-1, VCAM and ICAM	
Cocoa powder (40 g/250 mL M or W)	AI (6 h)	CO	Daily: 40.41 mg (+)-catechin	Before/After Cocoa (CM+CW) *vs*. W (Ctrl) CM *vs*. CW	18	Healthy	2 h plasma (BM) 6 h urine (BM)	↓NF-κB ↓E-selectin ↓ICAM-1 No effect: VCAM-1	[203]
			46.08 mg (−)-epicatechin						
			36.54 mg procyanidin B2						
			495.2 mg tot.PPh						
			425.7 mg tot.Pr						

AI—acute intervention; BM—biomarkers for dietary compliance (e.g., phase II epicatechin metabolites and/or total epicatechin or total flavanols); C—cocoa; CO—crossover design; CRP—C-reactive protein; hs-CRP—high sensitivity C-reactive protein; Ctrl—control; DC—dark chocolate; ICAM-1, Intercellular Adhesion Molecule-1; IL-6, Interleukin-6; M—Milk; MCP-1—Monocyte Chemoattractant Protein-1; MTI—medium-term intervention; NC—no control design; PA—parallel arm design; PPh—polyphenols; STI—short-term intervention; VCAM-1—Vascular Cell Adhesion Molecule-1; VLA-4—Very Late Activation Antigen-4; W—water; WC—white chocolate.

The differences in the circulating levels of cell adhesion molecules may reveal a stage for pathophysiological events due to activation or damage to various cells [204,205,206]. Some studies with solid designs (crossover and parallel arm), involving both healthy and unhealthy subjects, were not able to detect cocoa intake-provoked changes in the circulation of key adhesion molecules such as ICAM-1 and VCAM-1 [156,195,198,201]. However, some other studies reported a positive impact of cocoa consumption on the different types of CVD inflammation-related adhesion molecules. Thus, in a group of postmenopausal hypercholesterolemic women consuming a high-flavanol cocoa beverage (446 mg of total flavanols), there was a 2.4-fold increase in hyperemic blood flow associated with a significant decrease in plasma levels of VCAM-1, compared to the low-flavanol cocoa beverage group (43 mg of total flavanols). However, no differences were seen for E- and P-selectins, along with ICAM-1 [200]. On the other hand, no significant changes were observed for VCAM-1, but circulating ICAM-1 concentrations significantly decreased after consumption of dark chocolate (41 g/day) by healthy subjects [193]. A study performed by Monagas and colleagues suggests that the chronic consumption of cocoa powder may modulate the expression of adhesion molecules (soluble and expressed on T-cell and monocyte surfaces) related to the early stages of atherosclerosis in subjects at high risk of coronary heart disease. In a randomized crossover medium-term feeding trial they demonstrated a lower serum concentration of P-selectin and ICAM-1 and decreased expression of cell adhesion molecules (very late activation antigen-4 (VLA-4), CD40, and CD36) on the monocyte surface after intervention with cocoa powder in skimmed milk (C + M) *vs*. only skimmed milk ingestion (M). The circulating levels of VCAM-1 and MCP-1 concentrations were also lower, but not statistically significant, while that of E-selectin remained unchanged after C+M compared with M intake [123]. Their results agreed with the previously reported decrease in ICAM-1 in healthy volunteers due to chronic dark chocolate consumption [193]. However, for the first time a positive effect of cocoa consumption on P-selectin concentrations was reported by Monagas and colleagues, in contrast to previous results obtained on both healthy [176] and health-compromised subjects [198,200].

Nuclear factor κB (NF-κB) is a key molecule in the pathophysiology of atherosclerosis involved in the regulation of adhesion molecules and cytokine expression [207]. A recent crossover study on healthy subjects evaluated the effect of acute cocoa consumption in different matrices related to the bioavailability of cocoa polyphenols in NF-κB activation and the expression of adhesion molecules [203]. The administration of 40 g of cocoa powder either with milk (CM) or water (CW) decreased ICAM concentration 6 h after intake, while E-selectin levels were lower only after CW intervention, with no changes in VCAM-1 concentration either with CM or CW interventions. Acute consumption of cocoa with water (CW) significantly decreased NF-κB activation in peripheral blood monocytes compared to baseline and to CM [203], whereas milk treatment alone (control) significantly induced NF-κB activation. On the other hand, cocoa mixed with milk had no effects on NF-κB activation. The observed differences were attribute to the different bioavailability of polyphenolic compounds present in cocoa powder when taken with milk or water, which was monitored during the study as urinary host phase II (epicatechin-Glucs and -Sulfs) and microbial (sum of the 3,4-dihydroxyphenylacetic, protocatechuic, 4-hydroxybenzoic, 4-hydroxyhippuric, hippuric, caffeic and ferulic acids) metabolites [203]. Therefore, it was suggested that cocoa consumption could confer beneficial anti-inflammatory effects mediated by inhibition of the NF-κB-dependent transcription pathway or interaction with certain cytokines and the food matrix can modulate this effect.

Interleukins are thought to be involved in the chronic inflammatory response that is typical of atherosclerosis [206]. A one-week regular consumption of dark chocolate (100 g/day) in normal weight obese women showed a significant reduction in the serum level of the interleukin-1 receptor antagonist (IL-1Ra) and its positive correlation with total cholesterol, LDL cholesterol and CVD risk indexes changes. A neutral effect on other pro-inflammatory cytokine (IL-1α, IL-1β, IL-6, and TNF-α) and hs-CRP levels was reported [166]. Similar results were obtained for inflammatory markers including TNF-α, IL-1β, IL-6 and P-selectin along with hs-CRP in a non-controlled clinical trial, which failed to find any changes in interleukins in healthy subjects upon consumption of polyphenol-rich cocoa supplements, consisting of dark chocolate (36.9 g/day) and cocoa powder (30.95 g/day) for 6 weeks [176]. However, a recent crossover study [156] on normo- and hyper-cholesterolemic subjects demonstrated the decreased serum level of IL-1β and IL-10 after regular 4-week consumption of cocoa products with milk (15 g twice per day) as compared to control (milk). Other measured parameters such as IL-6, TNF-α, IL-8, MCP-1, and vascular and intracellular cell adhesion molecule levels remained unchanged [156].

Recently, leukotrienes (LTs) have been implicated as mediators, biomarkers, and possible therapeutic targets in the context of subclinical atherosclerosis [208]. Leukotrienes are arachidonic acid (AA)-derived lipid mediators of inflammation, exerting a range of pro-inflammatory effects, and have proved to be important mediators in inflammatory conditions such as preclinical atherosclerosis. For this reason, it has been suggested that leukotriene synthesis inhibitors and leukotriene receptor antagonists induce beneficial effects at preclinical stages of the atherosclerotic process [209]. Data from an early short-term crossover intervention study suggest that cocoa polyphenols can favourably alter eicosanoid synthesis in humans, providing a plausible hypothesis for a mechanism by which they can decrease platelet activation in humans [89]. Thus, the consumption of high-procyanidin chocolate resulted in increased prostacyclin and decreased leukotriene concentrations, and, as result, in a decreased leukotriene-prostacyclin ratio, a measure of the pro-inflammatory/anti-inflammatory eicosanoid balance [89].

Although the majority of human studies provide evidence for a possible interaction between cocoa polyphenols and inflammatory mediators involved in CVD, practically all of them warn about certain limitations and appeal for further, better designed intervention studies to confirm the presented outcomes. Overall, in order to determine the biological effect of cocoa polyphenols and to reach any beneficial conclusion that could possibly be implemented in human health, large-scale randomized placebo-controlled studies are required to confirm and expand upon the potential anti-inflammatory role of polyphenol-rich cocoa products. Moreover, the design of human intervention trials should use a relevant amount of cocoa products, which corresponds to real-life doses and could readily be incorporated into the regular human diet.

### 4.2. Animal Models, *in Vitro* and Cell Culture Studies

There are not many *in vivo* animal studies specifically focused on the CVD-related activity of cocoa polyphenols, and even fewer considering inflammatory mediators [41]. However, as demonstrated by human studies, animal intervention studies also support the capability of cocoa polyphenols to effectively suppress the production of cytokines and adhesion molecules that promote CVD-related inflammation. In a recent study by Gu and colleagues [210], male mice previously fed with a high-fat (HF) diet for 8 weeks were randomized to a HF diet or HF diet supplemented with 8% cocoa powder (HF–HFC group) for 10 weeks. Cocoa supplementation significantly decreased the plasma levels of the pro-inflammatory mediators, IL-6 (30.4%) and the expression of several pro-inflammatory genes (*Il6*, *Il12b*, *Nos2*, and *Emr1*) in mice. Moreover, unlike the study reported by Monagas and colleagues [123] in humans, the above study also demonstrated a significant decrease in MCP-1, after cocoa supplementation. 

It is noteworthy that the composition of chocolate or cocoa differing in polyphenol contents and dietary doses could lead to different outcomes in CVD risk end points [211,212]. Most of the human intervention studies using chocolate or cocoa powder supplementation have reported a protective effect related to systemic inflammation. On the other hand, a recent study by Yakala *et al*. [213] demonstrated the unfavorable effect of chocolate consumption on cardiovascular parameters. In this study, the effects of chocolate supplementation were studied in ApoE*3-Leiden mice, a model susceptible to diet-induced atherosclerosis, fed with a high-cholesterol control diet supplemented with two different chocolates (A and B), where chocolate A had a relatively high-polyphenol and low-fibre content compared to chocolate B. After both chocolate treatments, an increase in plasma cholesterol and atherosclerotic plaque formation was observed, compared to the mice fed only with a high-cholesterol diet. In addition, mice on a high-cholesterol diet supplemented with chocolate B showed elevated plasma levels of VCAM-1 and *E*-selectin, whereas the chocolate A showed no effects. Supplementation with chocolate A appeared to be less unfavorable than chocolate B with respect to inflammatory parameters, which was related to the higher circulating polyphenol concentrations present in the A group. Therefore, the discrepancies reported in this study were attributed to the difference in the polyphenol composition of chocolate and quantity consumed. In contrast to the chocolate intervention, a recent study reported that a 7-day high-cocoa diet (4.8 g/kg/day) reduced the production of pro-inflammatory cytokines (IL-6 and TNF-α), along with the production of NO and reactive oxygen species, in rat peritoneal macrophages *ex vivo* [214]*.* However, no significant changes were found in this study when a lower (2.4 g/kg/day) cocoa diet was applied.

In comparison to animal models, *in vitro* and cell culture models were more extensively applied in research on the CVD anti-inflammatory properties of cocoa polyphenols.

Table 2 represents the studies regarding the effects of cocoa polyphenols on inflammatory mediators discussed previously for human intervention studies. The *in vitro* and cell culture experiments were originally appointed to help identify cellular and molecular targets for the anti-inflammatory activities of cocoa polyphenols.

**Table 2:** *In vitro* studies on cocoa polyphenol CVD-related anti-inflammatory activities. Ref. = Reference.

Model	Treatment (dose)	Outcomes	Ref.
*Leukotrienes*			
(a) Isolated rabbit 15-LOX-1	(a) Cocoa procyanidins: monomers to decamers (2.9 mg/mL)	Dose-dependent: (a) ↓15-LOX-1 activity	[215]
(b) Recombinant human platelet 12-LOX	(b) Epicatechin & procyanidin decamers	(b) ↓12-LOX activity	
Recombinant human 5-LOX	Cocoa epicatechin & procyanidins	↓5-LOX activity	[216]
	(10 μmol/L)	↓Pro-inflammatory mediators (LTB4, LTC4, LTD4)	
*Pro-Inflammatory and Anti-Inflammatory Cytokines*			
PHA-stimulated PBMC	Cocoa procyanidins: monomers through decamers (25 μg/mL)	↓IL-1β secretion (monomer to tetramer)	[217]
		↑IL-1β secretion (pentamer to decamer)	
		↓IL-2 expression (pentamer to heptamer)	
		↓IL-4 expression & secretion (pentamer to decamer)	
Human PBMC	Cocoa procyanidins: monomers through decamers (25 μg/mL)	↑IL-1β transcription & secretion (pentamers-decamers)	[218]
		↓IL-1β transcription & secretion (monomers-tetramers)	
Resting and (PHA)-stimulated human PBMC	Cocoa procyanidins: monomers through decamers (25 μg/mL)	Resting PBMCs:	[219]
		↑IL-4 secretion (hexamer-decamer fraction)	
		PHA-stimulated PBMCs:	
		↑IL-4 secretion (monomeric fraction)	
		↓IL-4 secretion (hexamer-decamer fraction)	
Resting and (PHA)-stimulated human PBMC	CFP fractions: monomers through decamers (25 μg/mL)	↓ TNF-α (monomers and dimers)	[220]
		↑ TNF-α (tetramers through octamers)	
PHA-stimulated PBMC	Cocoa flavanols and their related oligomers (25 μg/mL)	↓ IL-5 release (oligomeric fractions: hexamers to decamers)	[221]
Rat NR8383 macrophages	Cocoa polyphenol extract (10–50 μg/mL tot. PPh) *vs*. epicatechin (30–60 μg/mL tot. PPh)	↓ TNF-α, MCP-1, IL-1α, IL-6)	[222]
Murine EL4BOU6 lymphocytes	Cocoa extract (5–80 μg/mL tot. PPh) *vs*. epicatechin (60–120 μg/mL tot. PPh)	↓IL-2 secretion	[223]
		↑IL-4 secretion	
		↓T lymphocyte activation	
Human PBMC	Cocoa flavanol fractions: Short (monomers-pentamers)	↑IL-1β, IL-6, IL-10, TNF-α (long-chain fraction)	[224]
	Long-chain (hexamers-decamers) (20 μg/mL)		
LPS-stimulated human PBMC	Cocoa phenolic acids: (3,4-DHPPA, 3-HPA,	↓TNF-α	[225]
	3,4-DHPAA, 3-HPAA,	↓IL-6	
	4-HBA) (1 µM)	↑IL-1β	
*NF-κB Activity*			
Jurkat T cells	Purified cocoa: [(−)-epicatechin (EC) and	↓NF-κB	[226]
	(+)-catechin (CT)] and a B dimeric procyanidin (DP-B) (1.7–17.2 µM)	↓IL-2 transactivation	

4-DHPAA—3,4-dihydroxyphenylacetic acid; 3,4-DHPPA—3,4 dihydroxyphenylpropionic acid; 3-HPA—3-hydroxyphenylpropionic acid; 3-HPAA—3-hydroxyphenylacetic acid; 4-HBA—4-hydroxybenzoic acid; CFP—Cocoa flavanols preparation; LOX—lipoxygenase; MCP-1—monocyte chemoattractant protein-1; PBMC—peripheral blood mononuclear cells; PHA—phytohemagglutinin; VSMC—vascular smooth muscle cells.

It has been shown that cocoa polyphenols can inhibit lipoxigenase activity *in vitro* [215,216]. Inflammatory mediators, leukotrienes, are formed via the 5-lipoxygenase pathway of arachidonic acid metabolism; therefore, cocoa polyphenols might modulate leukotrienes via eicosanoid metabolism [180]. Schewe and colleagues [215,216] reported that cocoa (−)-epicatechin and its low-molecular procyanidins inhibit both dioxygenase and LTA(4) synthase activities of human 5-LOX, the first two consecutive steps in the conversion of arachidonic acid into leukotrienes. Moreover, epicatechin and procyanidin decamer inhibited the recombinant human platelet 12-LOX. They suggested that this mechanism may underlie a putative anti-inflammatory effect of cocoa products. Thus, their outcomes are in line with the results from the *in vivo* study, where healthy subjects had lower levels of the plasma leukotrienes LTC4, LTD4 and LTE4, along with increased levels of prostacyclin, 2 h after procyanidin-rich chocolate consumption [89].

Intervention studies suggest that dietary cocoa polyphenols may modulate atherogenic inflammatory processes via interaction with cytokines. Several *in vitro* studies have studied the immunomodulatory effects of cocoa polyphenols on the production of cytokines in human peripheral blood mononuclear cells (Table 2). Thus, studies performed by Mao and colleagues [217,218,219,220,221] demonstrated that some fraction of cocoa polyphenols may exhibit anti-inflammatory activities by modulating the production of pro-inflammatory cytokines, including IL-1β, IL-2, IL-6 and TNF-α, and secretion of the anti-inflammatory cytokine IL-4 [219]. The nature and extent of this modulation were strongly dependent on the degree of polymerization of the tested procyanidins. Thus, smaller fractions of cocoa polyphenols (monomers-tetramers) consistently decreased pro-inflammatory IL-1b expression, while the larger oligomers (pentamers-decamers) increased its expression [218]. Ramiro and colleagues also reported that cocoa extract and epicatechin mediated a decrease in the secretion and RNA expression of various pro-inflammatory mediators by macrophages, such as MCP-1, TNF-α, IL-1α, and IL-6 mRNA expressions, suggesting that a cocoa polyphenol inhibitory effect on cytokine secretion is produced, in part, at the transcriptional level [222]. However, a striking increase in the LPS-induced synthesis of IL-1β, IL-6, IL-10, and TNF-α in the presence of long-chain flavanols from cocoa by mononuclear cells was demonstrated in another *in vitro* study [224]. Epicatechin and cocoa extract were also shown to significantly reduce IL-2Rα (CD25) expression and IL-2 secretion on activate peripheral blood mononuclear cells [223]. In resting and PHA-stimulated PBMC, the intermediate-sized cocoa flavanol and procyanidin fractions (tetramers to octamers) were shown to display an increase in TNF-α secretion. The monomers and dimers were slightly inhibitory, while trimers, nonamers and decamers induced TNF-α levels [220]. Cocoa flavanols and their related oligomers were also able to modulate IL-5 in PHA-stimulated peripheral blood mononuclear cells [221]. 

A study reported the effect of cocoa microbial-derived phenolic acids (3,4 dihydroxyphenylpropionic acid (3,4-DHPPA), 3-hydroxyphenylpropionic acid, 3,4-dihydroxyphenylacetic acid (3,4-DHPAA), 3-hydroxyphenylacetic acid, 4-hydroxybenzoic acid and 4-hydroxyhippuric acid (4-HHA)) on the modulation of the production of pro-inflammatory cytokines, *i.e.*, TNF-α, IL-1β and IL-6, in LPS-stimulated PBMC [225]. The phenolic acids used were at a biological concentration level (1 mM) within the range (0.1–10 mM) found in plasma samples after cocoa polyphenol intake [227]. Only the dihydroxylated compounds, 3,4-DHPPA and 3,4-DHPAA, with the exception of 4-HHA for TNF-α secretion, significantly inhibited the secretion of these pro-inflammatory cytokines. The concentrations of IL-6 were reduced with 3,4-DHPPA and 3,4-DHPAA pretreatment. This study demonstrated that dihydroxylated phenolic acids derived from colonic microbial metabolites could probably act as an anti-inflammatory agent, providing favourable effects on CVD [225].

*NF-κB* is a redox-sensitive transcription factor that regulates the expression of large family of genes, including those encoding proteins involved in inflammation such as IL-1, IL-6, and TNF-α [228]. Flavanols have been shown to modulate the inflammatory effect in cultured RAW264.7 murine macrophages via NF-κB pathways, and this depended on the degree of polymerization [229]. Thus, monomers and dimers repressed the TNF-α secretion and NF-κB-dependent gene expression induced by interferon γ, whereas the procyanidin C2 enhanced them. Epicatechin monomers were also shown to interact with NF-κB and inhibit TNF-α-stimulated activation of T lymphocytes [226]. Epicatechin and procyanidins can inhibit NF-κB at different levels in the activation pathways, where the decrease in cell oxidants that are involved in NF-κB activation and the prevention of NF-κB activation via specific interaction with signaling proteins are seen as main mechanisms [230]. On the whole, the results from these studies demonstrate that cocoa polyphenols can act as modulators of the immune response in immune cells involved in the early stages of atherogenic inflammation.

Although only a few studies have been carried out, animal experiments are expected to contribute much to the mechanistic understanding of the role of cocoa polyphenols in CVD inflammatory processes. However, these models have certain limitations that should be considered while interpreting results and extrapolating them to humans: type and dose for intervention, model *vs.* human-specific metabolism, etc. Regarding *in vitro* and cell culture studies, the main concern is the type of cocoa polyphenols applied for treatment. Since in CVD-related inflammatory research models usually represent the processes normally occurring close to systemic circulation (immune cells, vascular and endothelial tissues), the main compounds considered in these interactions should be bioavailable metabolites of polyphenols, mainly presented as phase II host and microbial metabolites of epicatechin. In addition, it is difficult to make an alignment between doses of polyphenols tested in *in vitro* models and those normally related to dietary consumption in humans.

## 5. Conclusions

The data from clinical and experimental studies considered in this review suggest that cocoa products, due to its high polyphenol content, may exert anti-inflammatory properties. Yet much remains to be elucidated on the interaction of cocoa polyphenols with inflammatory mediators involved in cardiovascular pathology. Larger, well-designed, placebo-controlled studies are expected to approach this question. At this point, there is a certain need to address recently raised new aspects regarding cocoa polyphenol bioavailability in future investigations. One of them is the role of cocoa polyphenol metabolites in respect of their anti-inflammatory properties. Cocoa polyphenol bioavailability depends on numerous varying components, which are sometimes difficult to control in clinical and population studies. To get a better approximation of the association between cocoa polyphenol consumption and its health outcomes, a detailed monitoring of cocoa polyphenol bioavailability during intervention studies is required. Two main groups of metabolites should be considered, both in human and in model studies: host and microbiota-derived metabolites. Recent human studies have already started to account for these metabolites, not only as dietary compliance biomarkers, but also to assess their impact on human health [156,166,202,203,210,214]. This is also supported by the fact that polyphenol microbial metabolites can exert various biological activities, some of them with anti-inflammatory potential [106,123,225]. The role of phase II conjugation is another issue to be considered, especially in mechanistic studies on cocoa polyphenol bioactivities, e.g., their anti-inflammatory properties. Both host and microbial metabolites are mainly present as phase II conjugates in systemic circulation upon dietary intake of cocoa products. Therefore, they are principal forms of cocoa polyphenols available for targeting the inflammatory process *in vivo*. Being distinct from core compounds in their properties, these metabolites warrant being considered as principal mediators in cocoa CVD-related anti-inflammatory activities. On the other hand, a possible intracellular deconjugation metabolism of phase II metabolites, as was recently reported in a case with resveratrol [231], should also be considered and investigated in detail for cocoa polyphenols.
